# Third generation sequencing transforms the way of the screening and diagnosis of thalassemia: a mini-review

**DOI:** 10.3389/fped.2023.1199609

**Published:** 2023-07-06

**Authors:** Lixia Zhan, Chunrong Gui, Wei Wei, Juliang Liu, Baoheng Gui

**Affiliations:** ^1^ The Second School of Medicine, Guangxi Medical University, Nanning, China; ^2^ Child Healthcare Department, The Second People's Hospital of Beihai, Beihai, China; ^3^ Center for Medical Genetics and Genomics, The Second Affiliated Hospital of Guangxi Medical University, Nanning, China; ^4^ The Guangxi Health Commission Key Laboratory of Medical Genetics and Genomics, The Second Affiliated Hospital of Guangxi Medical University, Nanning, China

**Keywords:** third-generation sequencing, nanopore sequencing, single-molecule real-time sequencing, thalassemia, screening, diagnosis

## Abstract

Thalassemia is an inherited blood disorder imposing a significant social and economic burden. Comprehensive screening strategies are essential for the prevention and management of this disease. Third-generation sequencing (TGS), a breakthrough technology, has shown great potential for screening and diagnostic applications in various diseases, while its application in thalassemia detection is still in its infancy. This review aims to understand the latest and most widespread uses, advantages of TGS technologies, as well as the challenges and solutions associated with their incorporation into routine screening and diagnosis of thalassemia. Overall, TGS has exhibited higher rates of positive detection and diagnostic accuracy compared to conventional methods and next-generation sequencing technologies, indicating that TGS will be a feasible option for clinical laboratories conducting in-house thalassemia testing. The implementation of TGS technology in thalassemia diagnosis will facilitate the development of effective prevention and management strategies, thereby reducing the burden of this disease on individuals and society.

## 1. Introduction

Thalassemia is a group of inherited hemolytic anemia diseases resulting from genetic mutations that lead to the absence or deficiency of the synthesis of one or more globin chains in hemoglobin. The clinical symptoms of thalassemia were initially described by Dr. Thomas B. Cooley in 1925 (1). In 1936, Whipple and Bradford created the term “thalassemia” for this anemia disease, deriving it from the Greek words “thalassa”, refering to “sea”, and “haima”, meaning “blood”, as it was primarily prevalent in anemic patients from Mediterranean countries (2). Subsequently, the disease was found to be widespread in the Middle East, India, and Southeast Asia.

Thalassemia is classified into two main types based on the affected globin chains: α- and β-thalassemia. Clinically, thalassemia can be further classified into the following types: (1) Thalassemia major: It is the most severe form of thalassemia, characterized by severe anemia, growth retardation, hepatosplenomegaly, and skeletal abnormalities. (2) Thalassemia minor: It is a relatively mild form of thalassemia, and patients may only have mild anemia and minor clinical symptoms. (3) Thalassemia intermedia: Patients exhibit a higher degree of anemia compared to thalassemia minor but less severe than thalassemia major. Patients with thalassemia major usually require regular blood transfusions to replenish healthy red blood cells to relieve the symptoms of anemia. However, prolonged transfusions may lead to iron overload and require iron chelation therapy to prevent iron toxicity.

Currently, Hematopoietic Stem Cell Transplantation (HSCT) is a potentially curative treatment for thalassemia by transplanting normal hematopoietic stem cells into the patient to replace the damaged hematopoietic system. However, HSCT requires a matched donor and carries certain risks and complications. Recently, there have been significant advancements in gene therapy and gene editing approaches for thalassemia treatment. Notably, in August 2022, the U.S. Food and Drug Administration (FDA) approved Zynteglo, a gene therapy developed by bluebird bio incorporation, for the treatment of β-thalassemia patients. However, Zynteglo has gained attention due to its high cost, priced at $2.8 million, making it the most expensive drug on the market. Moreover, on June 8th, 2023, the FDA accepted Biologics License Applications for exagogene autotemcel (excel cel), the first medical product of CRISPR gene-editing, for severe transfusion-dependent β-thalassemia.

Considering the cost of treatment, thalassemia is still a significant social and economic burden, especially in highly endemic areas, with an estimated world prevalence of 5%–7% carriers and an annual birth rate of over 2.4% (3). In China, there are around 30 million thalassemia carriers and about 300,000 patients with severe and intermediate forms, with an increasing rate of approximately 10% per year (4). Highly endemic areas in China include Guangxi, Guangdong, Hainan, Yunnan, Sichuan, Hunan, and Jiangxi, with a population carriage rate of 1%–23% (4). Therefore, implementing screening programs for thalassemia is crucial to prevent new cases and maintain the desired annual birth rate of thalassemia major, decreasing lifetime maintenance costs for patients.

Traditional thalassemia screening involves a three-step workflow, starting with a full blood count (FBC) and erythrocyte morphology to observe and calculate mean corpuscular volume (MCV) and mean erythrocyte hemoglobin content (MCH) to identify low hemoglobin levels and abnormal erythrocyte content. Subsequent biochemical analysis is performed using hemoglobin (Hb) electrophoresis, high-performance liquid chromatography (HPLC), or capillary electrophoresis (CE). Confirmatory tests such as spanning break site assay ((gap-polymerase chain reaction, Gap-PCR)), reverse spot hybridization assay ((polymerase chain reaction-reverse dot blot, PCR-RDB)), multiplex linkage probe amplification (MLPA), or conventional genetic tests like direct Sanger sequencing are then used in basis of preliminary results. While these methods are considered the gold standard for thalassemia investigations, they are labor-intensive, and over 1,530 hemoglobin-related genomic variants have been identified to date, which can further complicate the interpretation of thalassemia, especially in the presence of abnormal hemoglobin genotypes or modifier genes. Moreover, traditional methods are not reliable in accurately diagnosing rare mutations, which necessitates the development of new DNA screening tools.

**Figure 1:**
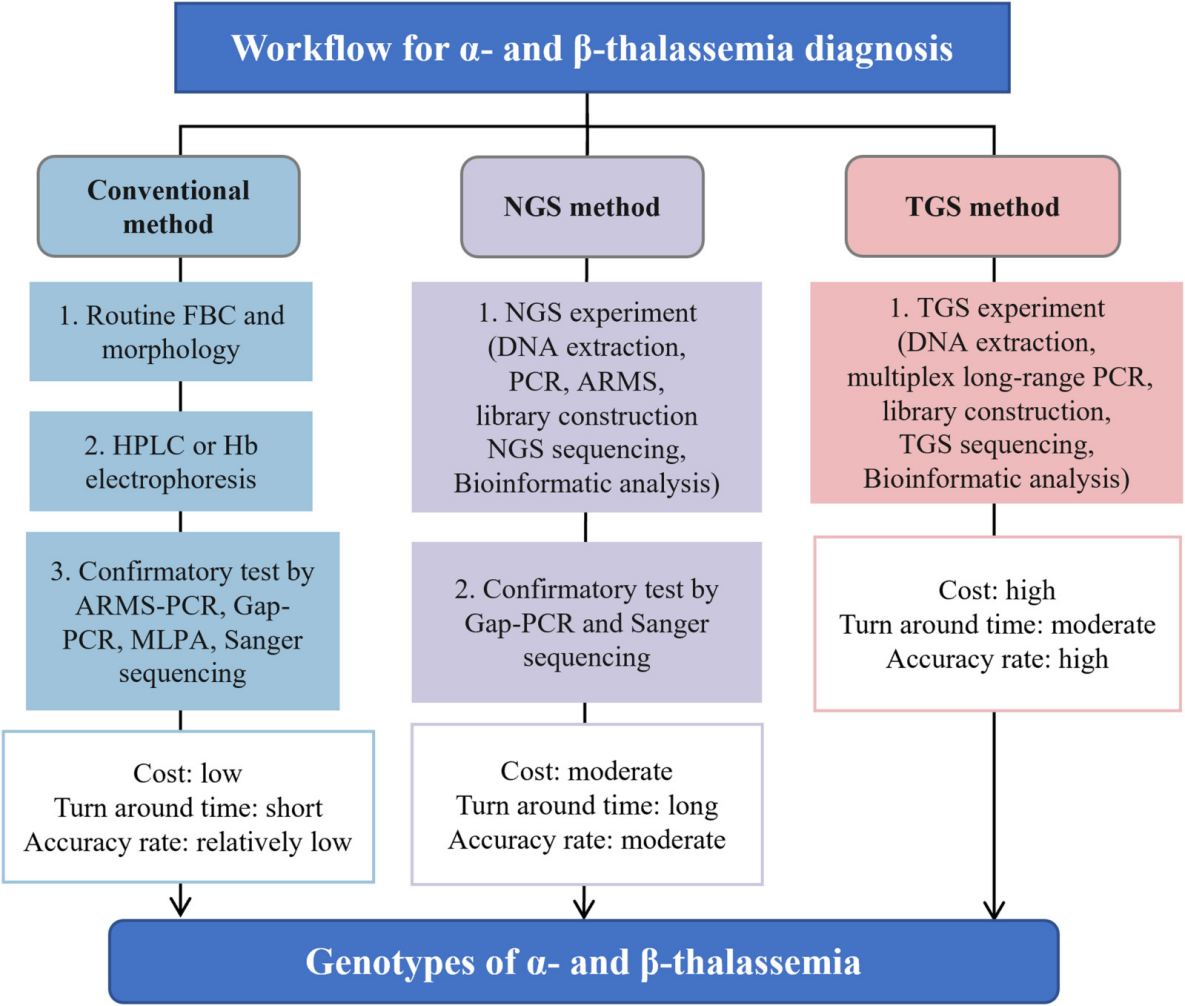
The general process flow, advantages and disadvantages for screening and diagnosing thalassemia using conventional, NGS and TGS approaches. Figure adapted from Suhaimi et al. (5). TGS, third-generation sequencing; NGS, next-generation sequencing; FBC, full blood count; PCR, polymerase chain reaction; ARMS, amplification refractory multiple sequencing; HPLC, high-performance liquid chromatography; MLPA, multiplex ligation-dependent probe amplification.

Next-generation sequencing (NGS), also known as second-generation sequencing (SGS), has found wide application in clinical practice for its ability to identify mutations across the whole human genome, which can reveal various genetic diseases. However, there are several limitations that hinder its application as a stand-alone technology for screening and diagnosing thalassemia, requiring the aid of Gap-PCR and Sanger sequencing (Figure 1) (5). Third-generation sequencing (TGS) has emerged as an alternative method that demonstrates great potential for thalassemia detection. This article aims to describe the latest and most widespread uses, advantages of TGS technology for thalassemia detection, as well as the challenges and solutions associated with its incorporation into standard screening and diagnostic protocols.

## 2. Overview of third-generation sequencing

TGS is a newer sequencing technology and has rapidly advanced the field of genomics. TGS technologies differ from traditional second-generation sequencing (SGS) methods by facilitating direct sequencing of long-stranded DNA or RNA molecules without fragmentation or amplification. TGS technologies mainly include single-molecule real-time (SMRT) sequencing, nanopore sequencing, and synthetic long-read (SLR) sequencing.

The earliest TGS technology, SMRT sequencing, was developed by Pacific Biosciences (PacBio) and was introduced in 2010. SMRT sequencing detects nucleotide binding as the DNA polymerase travels along a single DNA molecule, producing long reads of up to tens of kilobases with high accuracy and low error rates. In 2014, the introduction of nanopore sequencing by Oxford Nanopore Technologies (ONT) allowed for the detection of changes in current as DNA or RNA molecules pass through a nanopore, resulting in a new sequencing technology. This technology also produces long reads, with an average length of tens of kilobases, and can be used for real-time sequencing applications. In recent years, several other TGS technologies have been developed, including synthetic long-read sequencing. SLR developed by Illumina combines short-read sequencing and long-range PCR to produce synthetic long reads.

TGS technologies have revolutionized genomics research by allowing the detection of complex genomic changes that were previously challenging or impossible to identify. TGS technologies have also enabled the sequencing of long-read transcriptomes, metagenomes, and epigenomes, broadening the scope of genomic research. TGS technology has a few clinical applications, including the diagnosis and management (e.g., 6–9).

## 3. The application of TGS in thalassemia

As a novel sequencing technology, the use of TGS in thalassemia is a recent development, with most reports published after 2020. A search of the authoritative literature database PubMed using keywords such as “thalassemia third-generation sequencing”, “thalassemia long read sequencing”, “thalassemia single-molecule real-time”, and “thalassemia nanopore” yielded a total of 30 relevant reports (https://pubmed.ncbi.nlm.nih.gov/; as of March 11, 2023). These studies demonstrate that TGS is gaining popularity as a technology for thalassemia screening and diagnosis, with 27 out of the 30 reports originating from China (refer to Table 1).

**Table 1:** Reposts using TGS in thalassemia screening and diagnosis from 2021 to 2023.

Hospital location	Application field	Number of subject test	Detection method	TGS platform	TGS efficacy finding	Reference and year
Hunan, China	PS	1,759	TGS vs. TM	PacBio	100% detection rate with higher genotype accuracy for individual carriers	Liang et al. 2021 (6)
–	PS	100	TGS vs. TM	PacBio	Detection of 10 more rare ground-poor mutations	Peng et al. 2022 (10)
Guangdong, China	PS	9	TGS vs. TM	PacBio	Detection more mutations and one-step determination of cis or trans conformation	Jiang et al. 2022 (11)
Fujian, China	PS	70	TGS vs. TM	PacBio	High detection rate of 7.14% in rare thalassemia and the first record in China with three variants found	Zhuang et al. 2022 (12)
Fujian, China	PS	33	TGS vs. TM	PacBio	The same detection rate of HKαα carriers, and detection of two more β-variants	Zhang et al. 2023 (13)
Guangxi, China	PS	434	TGS vs. TM	PacBio	Higher detection rate (83.18% vs. 73.27%) and more detection of rare genotypes	Luo et al. 2022 (14)
Guizhou, China	PS and prenatal screening	176 and 53 couples	TGS vs. TM	PacBio	Higher detection rate, and finding that five of couples had offspring at high risk of severe thalassemia	Wu et al. 2022 (15)
Hainan, China	PS	1,122	TGS vs. NGS	PacBio	Improved detection rates for common, rare and unknown genotypes	Huang et al. 2023 (16)
Guangdong, China	PS	23	TGS vs. NGS	PacBio	The same detection rate and correction of NGS misdiagnosis on 2 heterozygotes	Zhou et al. 2022 (17)
Guangdong, China	PGD	32	TGS vs. NGS	PacBio	Consistent with the NGS results on haplotype linkage analysis	Wu et al. 2022 (18)
Yunnan, China	PGD	5	TGS vs. NGS vs. TM	ONT	Consistent detection rates, but sequencing equipment is more affordable than NGS	Liu et al. 2021 (19)
Guangdong, China	NIPD	26	TGS vs. NGS	ONT	Haplotype typing was achieved in all families based on a 20 kb amplicon library	Jiang et al. 2021 (20)
Hunan, China	DRTV	278	TGS vs. TM	PacBio	Improved the detection rate by 19%, detected more rare genotypes, and corrected the diagnosis of 1 heterozygote by traditional methods	Liang et al. 2023 (21)
Guangxi, China	DRTV	32	TGS vs. TM	PacBio	Genotype detection rate of 100% and one-step differentiation of different genotypes	Li et al. 2022a (22)
Hubei, China	DRTV	2	TGS vs. NGS	PacBio	Diagnosis of a novel 10.3 kb deletion causing α^0^-thalassemia	Xu et al. 2023 (23)
Guangxi, China	DRTV	206	TGS vs. TM	PacBio	Same detection rate, and correction of 3 cases of misidentification based on traditional methods	Li et al. 2022c (24)
Guangxi, China	DRTV	4	TGS vs. TM	PacBio	Same detection rate and ability to directly identify the structure of genotypes	Long et al. 2022 (25)
Guangxi, China	DRTV	4	TGS vs. TM	PacBio	Diagnosis of four more rare mutations of thalassemia	Luo et al. 2022 (26)
Guangxi, China	DRTV	1	TGS vs. TM	PacBio	Diagnosis of a novel 4.9 kb deletion at beta-globin gene	Chen et al. 2022 (27)
Guangxi, China	DRTV	1	TGS vs. TM	PacBio	Diagnosis of a novel 107 kb deletion in the alpha-globin gene	Li et al. 2022c (28)
Hunan, China	DRTV	49	TGS vs. TM	PacBio	Diagnosis of eight more rare mutations of thalassemia	Liu et al. 2023 (29)
Guangdong, China	DRTV	1	TGS vs. TM	PacBio	Diagnosis of a novel 7.2 kb deletion at β-globin gene	Zhong et al. 2022a (30)
Guangdong, China	DRTV	1	TGS vs. TM	PacBio	Diagnosis of a novel 15.8 kb deletion at α-globin gene	Zhong et al. 2022b (31)
Guangdong, China	DRTV	1	TGS vs. TM	PacBio	Successful diagnosis of α^+^ thalassemia subtype −α^3.7III^	Bao et al. 2022a (32)
Guangdong, China	DRTV	1	TGS vs. TM	PacBio	Diagnosis of a novel 5 kb deletion at β-globin gene	Bao et al. 2022b (33)
Minnesota, USA	DRTV	4	TGS vs. TM	PacBio	Improving the identification of structural variants in complex β-globin gene cluster	Rangan et al. 2021 (34)
Guangxi, China	DRTV	1	TGS vs. TM	PacBio	Diagnosis of novel rearrangements of the α-globin gene cluster	Ning et al. 2022 (35)
Guangdong, China	DRTV	2	TGS vs. TM	PacBio	Conformation of Hb Q-Thailand heterozygosity not typically associated with the (−α^4.2^/) α^+^-thalassemia deletion allele	Qin et al. 2022 (36)
/	Editorial	/	TGS vs. TM	/	Improved sensitivity and specificity, with important advantages in the prenatal diagnosis	Toledo and Lafferty 2023 (37)
/	Review	/	TGS vs. NGS	/	Advantages in homologous gene variant discovery and copy number variation calling	Hasson et al. 2023 (38)

PS, Population screening; PGD, preimplantation genetic diagnosis; NIPD, non-invasive prenatal diagnosis; DRTV; diagnosis of rare thalassemia variants; TGS, third-generation sequencing; NGS, next-generation sequencing; TM, traditional method; PacBio, Pacific Biosciences; ONT, Oxford Nanopore Technologies; –, unavailable.

### 3.1. TGS in the molecular screening of thalassemia carriers

To reduce the incidence of thalassemia major infants, a mandatory mass screening strategy and appropriate genetic counseling are necessary for at-risk populations (39). Thalassemia screening using TGS has recently emerged (see Table 1). A more comprehensive and universal TGS screening framework, called Comprehensive Analysis of Thalassemia Alleles (CATSA), was established for thalassemia in 2021 (6). The CATSA method involves DNA extraction, PCR, library construction, sequencing, and bioinformatics analysis (Figure 1). A crucial step in the CATSA method is the utilization of a multiplex long-range PCR approach with primers optimized for specificity. This allows the generation of targeted amplicons that encompass all known thalassemia regions of insertions, deletions, single nucleotide variants (SNVs), structural variations (SVs) and copy number variations (CNVs). In the CATSA study, a total of 1759 primary screening samples with positive routine blood or hemoglobin tests were subjected to CATSA screening. The results were subsequently validated using double-blind testing and other “gold standard” methods, which demonstrated that the CATSA test achieved a 100% accuracy rate.

Several other studies have been conducted to validate the CATSA method for population screening, with most reporting improved detection rates compared to standard methods (refer to Table 1). For instance, Peng et al. (10) performed CATSA and conventional methods on 100 patients with suspected thalassemia and observed that CATSA identified 10 more rare clinical variants than the latter, including the −α^3.7III^ subtype, which was reported for the first time in China. The findings suggest that rare thalassemia variants are more prevalent than previously thought and are often misdiagnosed by conventional diagnostic methods. Liu et al. (29) conducted CATSA tests on 49 subjects who were negative for specially designed GAP-PCR and Sanger sequencing tests and identified mutations in the thalassemia gene in 8 subjects, indicating a higher positive detection rate of CATSA.

Screening of prospective parents for thalassemia carriers has received equally attention in many countries, especially in Israel, Sardinia and Cyprus, where mandatory premarital screening has been implemented in recent decades and through which the burden of thalassemia has been successfully reduced (40). Recently, TGS has also been applied to prenatal screening for thalassemia. In Wu et al. (15), 53 couples were examined for homozygous thalassemia mutations using TGS. The result showed that seven of them were identified as positive carriers, with five of them at risk of severe thalassemia during pregnancies, highlighting the potential of TGS for prenatal screening in regions with a high incidence of thalassemia.

### 3.2. TGS in the molecular diagnosis of thalassemia

Accurately determining the severity of a disease is a critical step in selecting the appropriate treatment, particularly in cases where patients exhibit atypical clinical manifestations or where conventional genotyping methods yield uncertain diagnoses. TGS technology, similar to NGS, can directly detect thalassemia genotypes and provide efficient interpretation of thalassemia variants. TGS has been successfully applied in various clinical scenarios, including preimplantation genetic diagnosis (PGD), non-invasive prenatal diagnosis (NIPD), and rare thalassemia diagnosis (as summarized in Table 1).

In the context of PGD, Liu et al. (19) evaluated the effectiveness of TGS using the Oxford Nanopore sequencing platform and observed consistent diagnostic outcomes with those obtained through conventional methods and NGS. Additionally, the cost of sequencing equipment was lower than that of NGS. Wu et al. (18) employed TGS to conduct haplotype linkage analysis on 32 embryos from three couples and succeeded in establishing haplotype linkage in 68.75% of them. These results align with those obtained via NGS, suggesting that TGS has promising potential for PGD applications. In a study examining NIPD applications, Jiang et al. (20) analyzed β-thalassemia in 13 families via TGS combined with relative haplotype dosage (RHDO) analysis using the Oxford Nanopore sequencing platform. The results indicated successful haplotyping in all 13 families when using a 20 kb amplicon library, demonstrating the feasibility of the TGS protocol. The advantages of TGS in detecting and interpreting thalassemia genotypes highlight its potential for improving the accuracy of diagnosis and facilitating personalized treatment strategies for patients with thalassemia.

## 4. Advantages of TGS in screening and diagnosis of thalassemia

Compared to traditional methods, TGS has shown significant advantages in thalassemia detection. TGS can cover over hundreds of α- and β-deletion variants in a single test, which surpasses the coverage of all previous methods for thalassemia detection. Moreover, TGS improves the detection rate of positive thalassemia genes, especially rare ones, by up to 19%. This can help avoid underdiagnoses caused by traditional methods. Additionally, TGS enhances the accuracy of diagnosis and can avoid misdiagnosis by traditional methods, especially for complex heterozygotes. TGS allows for the direct detection of new thalassemia genetic variants and precise identification of cis-trans variants in a “one-step” manner, without the need for family verification. This greatly facilitates the interpretation of thalassemia variants. Furthermore, TGS has a simple operation process and eliminates the need for multiple methods of cross-validation and inference, thus avoiding possible contamination from tedious steps.

Compared to NGS, recent studies with larger sample sizes indicate that the detection rate of TGS is higher, especially for deletion variants and heterozygotes (16, 17, 23). This is due to the significant advantage of TGS in read length, which is much longer than that of NGS, making it easier to detect deletion variants in thalassemia genes (23). Medical practitioners have started to recognize TGS as the preferred method over NGS for detecting long deletion variants in the thalassemia gene (Table 1) (23, 27, 28, 30, 31, 33). On the other hand, NGS faces difficulties in accurately sequencing regions with abnormal GC content and highly repetitive sequences, which can lead to missed mutations and false-negative results. In contrast, TGS can address this limitation due to its longer read length and less preference for GC content, leading to higher detection rates and diagnostic accuracy for thalassemia genes than NGS. Additionally, another advantage of TGS over NGS is its shorter turnaround time. While NGS strategies typically require additional confirmation through Gap-PCR and Sanger sequencing, which can prolong the overall testing process, TGS provides more rapid results (Figure 1).

## 5. Challenges and solutions of TGS in routine detection of thalassemia

The accuracy of TGS has been a longstanding topic of debate and improvement for many years, which has also raised doubts about its application in disease diagnosis. However, recent advancements have demonstrated that the accuracy of TGS sequencing can reach levels comparable to other sequencing technologies, such as Illumina sequencing. For instance, the earlier versions of PacBio sequencing platform produced high native error rates (ca. 13%) (41), but the current sequencing accuracy is high, especially high-fidelity (HiFi) reads generated by the Circular Consensus Sequencing (CCS) mode with an accuracy of up to 99.999% (42). Similarly, earlier ONT's equipment also had a high native sequencing error rate (5%–25%) (43), while ONT claims that the new version 10 flow cells can deliver up to 99.99% base-calling accuracy. Furthermore, using an amplicon sequencing approach combining unique molecular identifiers (UMIs) with ONT or PacBio CCS, the accuracy rate can be over 99.99% (44). Consequently, the accuracy of TGS will no longer pose a challenge for the proper detection of thalassemia.

Currently, the main platform for thalassemia detection using TGS is the PacBio platform (see Table 1). Despite its significant technical advantages, the high equipment costs and long turnaround times of PacBio limit its integration into routine screening and diagnostic procedures. While the total cost of preparing TGS libraries and sequencing reagents for each sample has dropped to below $20 (21), mainstream PacBio sequencing equipment is still expensive (e.g., the PacBio Sequel II device costs approximately $350,000), limiting its adoption by many medical institutions. If TGS library preparation samples are sent to biotech companies for sequencing, the overall testing cycle takes about 8 days (including sample transportation time) in China, whereas the testing cycle for traditional methods is no more than 3 days (21). It is expected that PacBio will release sequencing equipment that covers medium and low throughput with greatly reduced costs, and this equipment can fully meet the low throughput testing requirements for thalassemia currently. At that time, if medical institutions can purchase this equipment for in-house sequencing, they can eliminate transportation time to the company and arrange sequencing work promptly, thereby shortening the testing cycle.

An alternative solution to address the issues of high equipment costs and long turnaround times is to use the ONT platform for thalassemia detection. The ONT devices available on their official website range from the cheapest MinION at $1,000 to the most expensive GridION at $49,955 (as of March 27, 2023), which are significantly more affordable than PacBio devices, and thus more accessible to most medical institutions. On the other hand, ONT's turnaround time is greatly reduced. For example, a study reported a total turnaround time of 6 h for detecting viruses such as Ebola, Chikungunya and Hepatitis C virus (45). Although there is still limited research on the use of ONT in thalassemia detection, with only two studies available (see Table 1). Both the studies have demonstrated promising clinical potential for ONT in NIPD and PGD (19, 20). It is believed that in the near future, it will be a good alternative to traditional methods and facilitate the transition to third-generation sequencing technology for screening of thalassemia.

## 6. Conclusion

Third-generation sequencing (TGS) is a novel technology that has not yet been widely implemented for the screening and diagnosis of thalassemia in clinical practice worldwide. However, recent validation trials and preliminary studies have demonstrated that TGS using the PacBio platform provides significant advantages over traditional methods and NGS in terms of positive detection rate and diagnostic accuracy for thalassemia (see Table 1). Nevertheless, the high equipment cost and lengthy turnaround time currently limit the integration of PacBio-based TGS into routine thalassemia testing (summarized in Figure 1). Although ONT is a promising TGS platform with lower equipment cost and shorter turnaround time, its efficacy for thalassemia genetic testing has not been fully explored. Large-scale comparative trials are still required to validate its utility and accuracy. With the ongoing improvement in the reduced cost and faster turnaround time for PacBio, as well as the increased efficacy of ONT, it is anticipated that TGS will become a viable and cost-effective alternative for clinical laboratories to perform in-house thalassemia testing in the near future.
