# Correction: Third generation sequencing transforms the way of the screening and diagnosis of thalassemia: a mini-review

**DOI:** 10.3389/fped.2025.1637415

**Published:** 2025-07-02

**Authors:** Lixia Zhan, Chunrong Gui, Wei Wei, Juliang Liu, Baoheng Gui

**Affiliations:** ^1^ The Second School of Medicine, Guangxi Medical University, Nanning, China; ^2^ Child Healthcare Department, The Second People’s Hospital of Beihai, Beihai, China; ^3^ Center for Medical Genetics and Genomics, The Second Affiliated Hospital of Guangxi Medical University, Nanning, China; ^4^ The Guangxi Health Commission Key Laboratory of Medical Genetics and Genomics, The Second Affiliated Hospital of Guangxi Medical University, Nanning, China

**Keywords:** third-generation sequencing, nanopore sequencing, single-molecule real-time sequencing, thalassemia, screening, diagnosis

**A Correction on**
Third generation sequencing transforms the way of the screening and diagnosis of thalassemia: a mini-review By Zhan L, Gui C, Wei W, Liu J and Gui B (2023). Front. Pediatr. 11:1199609. doi: 10.3389/fped.2023.1199609

**Incorrect funding**

In the published article, there was an error in the Funding statement. [The current funding statement: “This work was supported in part by the National Natural Science Foundation of China (82001531 and 81860272 to BG), Guangxi Major Research Programme (AB22035013 to BG), Guangxi Natural Science Foundation (2023GXNSFBA026124 to CG, 2018GXNSFAA281067 to BG), Initial Scientific Research Fund for Advanced Talents from The Second Affiliated Hospital of Guangxi Medical University (2019112 to BG), Special Scientific Research Fund of Guangxi Ten-Hundred-Thousand Talents Project (2021186 to BG), and the Science Foundation for Young Scholars of Guangxi Medical University (GXMUYSF202115 to CG), Beihai Science and Technology Plan Project (2019D06 to LZ).”

Upon reviewing the final version of the article, we have identified an error in the order of the funds listed. The current order of the funds does not accurately reflect the primary sources of support for the research. Specifically, the Guangxi Major Research Programme (AB22035013 to BG) should be listed first, followed by the National Natural Science Foundation of China (82001531 and 81860272 to BG), and so on. The correct order of the funds are crucial for proper attribution and compliance with the funding agencies' requirements.]. The correct Funding statement appears below.

**Funding**

[This work was supported in part by the Guangxi Major Research Programme (AB22035013 to BG), National Natural Science Foundation of China (82001531 and 81860272 to BG), Guangxi Natural Science Foundation (2023GXNSFBA026124 to CG, 2018GXNSFAA281067 to BG), Initial Scientific Research Fund for Advanced Talents from The Second Affiliated Hospital of Guangxi Medical University (2019112 to BG), Special Scientific Research Fund of Guangxi Ten-Hundred-Thousand Talents Project (2021186 to BG), and the Science Foundation for Young Scholars of Guangxi Medical University (GXMUYSF202115 to CG), Beihai Science and Technology Plan Project (2019D06 to LZ)]

